# PRN Medicines Optimization and Nurse Education

**DOI:** 10.3390/pharmacy8040201

**Published:** 2020-10-26

**Authors:** Mojtaba Vaismoradi, Sue Jordan, Flores Vizcaya-Moreno, Ingrid Friedl, Manela Glarcher

**Affiliations:** 1 Faculty of Nursing and Health Sciences, Nord University, 8049 Bodø, Norway; 2 Department of Nursing, Swansea University, Swansea SA2 8PP, UK; s.e.jordan@swansea.ac.uk; 3 Nursing Department, Faculty of Health Sciences, University of Alicante, 03080 Alicante, Spain; flores.vizcaya@ua.es; 4 Hospital Graz II, A Regional Hospital of the Health Care Company of Styria, 8020 Graz, Austria; ingrid.friedl@kages.at; 5 Institute of Nursing Science and Practice, Paracelsus Medical University, 5020 Salzburg, Austria; manela.glarcher@pmu.ac.at

**Keywords:** education, medicines management, nurse, patient safety, *pro re nata*, PRN

## Abstract

Medicines management is a high-risk and error prone process in healthcare settings, where nurses play an important role to preserve patient safety. In order to create a safe healthcare environment, nurses should recognize challenges that they face in this process, understand factors leading to medication errors, identify errors and systematically address them to prevent their future occurrence. “*Pro re nata*” (PRN, as needed) medicine administration is a relatively neglected area of medicines management in nursing practice, yet has a high potential for medication errors. Currently, the international literature indicates a lack of knowledge of both the competencies required for PRN medicines management and the optimum educational strategies to prepare students for PRN medicines management. To address this deficiency in the literature, the authors have presented a discussion on nurses’ roles in medication safety and the significance and purpose of PRN medications, and suggest a model for preparing nursing students in safe PRN medicines management. The discussion takes into account patient participation and nurse competencies required to safeguard PRN medication practice, providing a background for further research on how to improve the safety of PRN medicines management in clinical practice.

## 1. Burden of Medication Errors

Adverse effects of medical treatment are a serious public health problem, and one of the leading causes of death worldwide [1,2,3]. Preventable harm affects 6% of patients (95% confidence intervals: 5–7%), and some 25% of incidents relate to medicines [1]. Medication errors affect all areas of healthcare, including hospitals, outpatient and general practice facilities, nursing homes, pharmacies and patient homes [2,4]. Within this paper, we use Aronson’s definition of a medication error, i.e.,

is an avoidable adverse effect of healthcare, whether or not it is obvious or harmful to the patient something incorrectly done through ignorance or inadvertence; a mistake, e.g., in calculation, judgement, speech, writing, action, or a failure to complete a planned action as intended, or the use of an incorrect plan of action to achieve a given aim [5] (p. 6013).

Not all medication errors are harmful, but they are often preventable [6,7]. An analysis of mortality rates by John Hopkins University over an eight-year period estimates that more than 250,000 deaths per year in the United States are attributable to healthcare errors [4], of which medication errors are the most common. Errors can occur multiple times during medicines management: this includes errors in prescribing, supplying, dispensing, preparing, administering or monitoring patients for medications’ side effects and adverse drug reactions (ADRs) [8]. Nevertheless, one third of all medication errors occur during medication administration [4]. Data from the European region show that the median percentages of hospital admissions due to ADRs and patients experiencing ADRs during hospitalization are 3.5% and 10.1%, respectively [9], and more serious ADRs often remain under-reported [10,11]. In healthcare systems, medication errors cause avoidable costs of between approximately €4.5 and 21.8 billion annually [12], and are important reasons for hospital admissions and mortality [13]. As such, healthcare organizations have the responsibility to establish a safety culture that focuses on improving the system and treats medication errors as challenges to be recognized and overcome [2]. Accordingly, a crucial step forward for the culture of safety in medicines management lies in the systematic identification and reporting of near-misses and errors during medication prescription, administration and follow up [14,15,16].

## 2. Nurses’ Roles in Medicines Management

As members of the health care team, nurses play a key role in medication safety. It is their job to prepare medication; calculate correct doses; and administer medication and monitor its effects, interactions or side effects, requiring up-to-date knowledge and skills relating to medicines management [17,18]. Nurses are directly involved in the care of patients and monitor the safety of medication before, during and after administration. It has been estimated that the medication administration procedure consumes up to 40% of the nurse’s time in a work shift [19]. They provide information and training to patients and give instructions on the safe use of medication during discharge [20]. Moreover, nurses, as mentors on clinical placements, play an essential role in training nursing students in safe medicines management [21].

On average, a nurse administers 10 doses of medication daily for each hospital patient [22]. Data from European Union Member States consistently show that medical errors and adverse events related to healthcare occur in 8% to 12% of hospital stays [23]. Increased potential for medication errors is evident in intensive care units (ICUs), where these are the most common type of error at 78% of cases. Critically ill patients admitted to intensive care units accumulate an average of 1.7 medical errors per day [24,25]. A study in the UK showed that 5796 medication errors happened over four data collection periods during 2014 and 2015 in various hospital wards [26]. Medication errors also occur frequently in long-term care, often due to lack of knowledge and competence, under-reporting of medication errors, confusion between trade-name products vs. generics, and work routines [27,28]. According to the results of a systematic review, 16–27% of nursing homes’ residents experienced medication errors. Although 75% of them were prescribed at least one potentially inappropriate medication, this remained underreported [29]. 

Nurses should gain required competencies in terms of knowledge and skills within regulatory, professional, legal and ethical frameworks regarding the prescription, storage, administration and safe disposal of medications. After receiving approprierte theoretical education and practical training, many nurses are authorized to prescribe medications from the national formulary within their scope of practice [30]. Therefore, they need to recognize the challenges they face in medicines management from prescription to administration, and follow-up of related side effects, and play their key role in the prevention of medication errors. They must be able to identify their own errors as well as those made by other healthcare providers, including pharmacists and physicians, and take appropriate preventive actions when administering medicines [14]. Additionally, they have to identify, report and manage errors committed by nursing students during their mentorship period [21,31]. It is therefore essential that factors contributing to errors are identified and systematically addressed in order to effectively improve patient safety. Such a perspective is in line with the World Health Organization’s (WHO) 3rd Global Patient Safety Challenge on Medication Safety. This challenge calls for creative initiatives to reduce severe, avoidable medication-associated harm in all countries by 50% over the next 5 years by all healthcare staff, including nurses, with the aim of reducing the length of hospital stays and the overall cost of healthcare, and improving patients’ well-being and satisfaction with care [32]. 

## 3. PRN Medication 

“*Pro re nata*” (PRN) is defined as the prescription of medications whose administration should be based on patients’ immediate needs rather than at predetermined administration times [33,34]. PRN prescription and administration is commonly used for psychotropic and psycholeptic medications, including antipsychotics (neuroleptics), anxiolytics, sedatives, and hypnotics, analgesics, gastro-intestinal preparations and other medicines used for relieving physical and psychological symptoms [34,35,36,37,38,39].

PRN medicines management is a crucial instrument in guiding the involvement of nurses working in a range of healthcare units, by which patients’ physical and mental sufferings are relieved based on nurses’ decision-making [28]. As front line staff in healthcare settings, nurses are located in the best position to systematically monitor patients’ needs, use their knowledge and skills and apply their authority regarding medication practice for making appropriate decisions on medicines management [40,41]. 

PRN prescription and administration aim to provide medicines as a complement to regularly scheduled medications, characterized by empowering both nurses and patients and providing flexibility for relieving physical and mental suffering and pain [42]. They are associated with an increase in the feeling of professional autonomy and a sense of self-worth and accomplishment for nurses in clinical practice [43]. Nonetheless, decision-making for PRN medicines by nurses is a complex task and is influenced by their pharmacotherapeutic competencies and skills, together with organizational routines and patient and family involvement [38]. The safety of PRN medicines management depends on close collaboration and partnerships between patients, nurses, physicians and pharmacists [44]. 

The high rate of PRN prescription and administration highlights the need for developing appropriate strategies for ensuring safe and appropriate PRN medicines management [37], especially given the current lack of relevant evidence-based clinical guidelines and instructions for practice at both national and international level [45]. PRN medicines form a significant proportion of medication errors, at between 9–40% in intensive care units; furthermore, PRN medications are especially likely to contribute to medical errors [34,46,47,48]. This is significant, given that over 90% of patients with psychiatric disorders receive at least one PRN medication [33]; 20–86% of nursing home residents receive some PRN drug administration [36,49]; and in outpatient clinics, the prevalence rate is about 77% [50]. However, more than one-third of patients are not monitored regarding their continuous needs for PRN medications after starting their prescriptions [35]. 

PRN medicines management has many benefits, but its inappropriate use can lead to polypharmacy, overdosing and over- or under-use, and administration without the patient’s consent or full disclosure of relevant information about medications [48]. Higher numbers of PRN prescriptions are associated with longer stays in nursing homes (median of 2.1 years), and polypharmacy (defined as ≥5 long-term medicines) [51]. 

Currently, many nursing students are not educated on PRN medicines management during degree courses, but studies have shown the effectiveness of nurses’ education on how to safely handle PRN medications’ prescription and administration [28]. Therefore, controversies surrounding PRN medicines management frequently consist of inappropriate assessment of patients’ outcomes, medications’ adverse side effects, and unclear documentation [52]. Crucially, there is an ongoing need for alignment of healthcare practice with clinical guidelines and improvements to the culture and practice surrounding PRN medications [53], together with standardization of PRN medicines management from prescription to administration and assessment of its side effects and any ADRs [54]. Clear educational strategies on PRN medication are also required, addressing how these are prescribed, administered and assessed in terms of their effects on the patients’ health conditions and care outcomes [48].

Nurse education is central to addressing these challenges: studies indicate the effectiveness of educational sessions for nursing staff on PRN medications in reducing the overall use of common medications including laxatives and hypnotics by 34–70% [55]. Empowering nurses through education clarifies the flow and coordination of activities, from medication prescription to follow-up and monitoring of side effects, by a multidisciplinary healthcare team [28,41]. It upgrades nurses’ positions in the process of patient care and medicines management, improves nurses’ involvement in medication safety and effectively motivates them to mobilize their theoretical knowledge and clinical judgment to achieve positive care outcomes. This in turn increases their job satisfaction and the feeling of autonomy, and consequently improves patient care outcomes [56,57]. However, there is a paucity of knowledge regarding appropriate education and training for nurses in PRN medicines management. We address this crucial oversight in the current literature by developing a model for these processes below.

## 4. A Model for Education in PRN Medicines Management 

**Figure 1:**
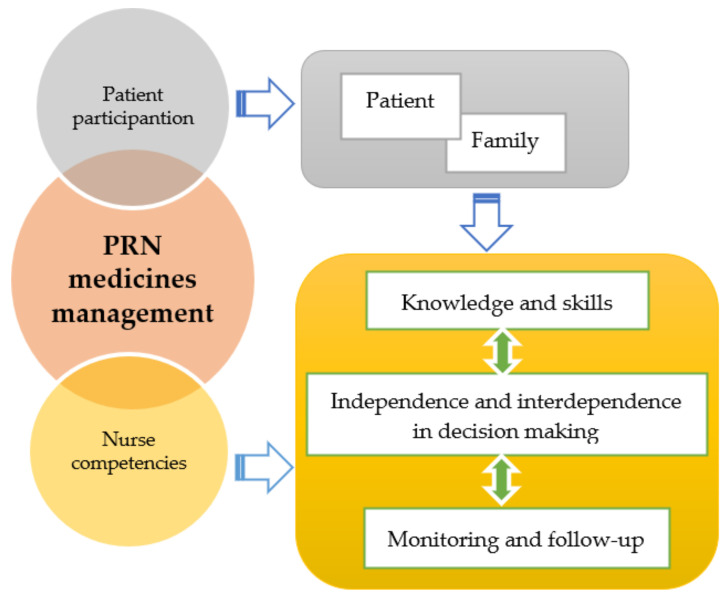
Model of the nursing education of *pro re nata* (PRN) medicines management.

Central to the identity and aims of PRN is a mutual, caring relationship between the patient and the nurse [34]: both of these actors have a mutual responsibility to ensure the safety of PRN [34,58]. Therefore, our suggested model (Figure 1) for the education of nursing students in ensuring safe PRN practice focuses on “patient participation” and “nurse competencies” as follows. 

### 4.1. Patient Participation

Nursing students should be educated regarding the significance of the patient’s role and participation in their own medicines management. They should be taught about educating patients and empowering them to monitor their own medication process in order to provide inputs about their needs as the basis for making decisions on PRN prescription and administration by nurses.

Patient education, as one side of the joint empowerment approach for both the healthcare provider and healthcare receiver, guarantees adherence to the principles of safe medication therapy [59]. This is characterized by a mutual understanding between the nurse and patient of the therapeutic reasons behind PRN medications’ prescription and administration to meet the real patient’s healthcare needs for medications [34]. Ideally, patients should be able to be trusted to perform a regular and unilateral evaluation of their medication process without healthcare providers’ participation [60]. If a patient is unable to take responsibility for their medication, relatives or informal caregivers can be educated and asked to manage the patient’s medication [61,62,63]. Informal and family caregivers can participate in the physical handling of PRN medication in terms of obtaining medications, preparing pillboxes or assisting with medication administration, organizing/tracking medications, collecting information and making treatment decisions [61], and educational activities as patient representatives [64]. Nevertheless, all reasonable measures should be used to encourage patients to take an active role [65,66]. 

**Figure 2:**
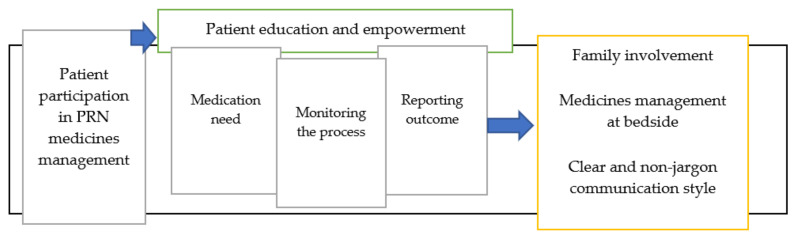
Patient participation in PRN medicines management.

Nursing students require training in communication skills, to meet expectations for engagement in PRN medication communication in line with a patient-centered approach to care [66] as has been depicted in Figure 2. They need to work from the standpoint that patients have the ability to learn about the medication process and can give meaningful reports on errors, near misses and discrepancies leading to patient harm [67,68]. It is crucial that nurses are able to give a voice to patients and invite them to become part of the decision-making process, through sharing information based on their literacy level and uncertainties [69]. The occasions of medication administration can be used to engage patients in medicines management, with an emphasis on informing patients regarding medications with implications for their own safety [70]. Patient requests to receive PRN medications should be considered a prompt for nurses to communicate with patients and inform them about the probable risks and benefits of medications along with their own role in the regular assessment and reporting of medications’ side effects [42]. The use of medical jargon and taking important medication decisions away from the patient’s bedside should be avoided [71]: this approach to care gives patients the feeling of ownership and control over medication administration and, by extension, their own physical and mental wellbeing [72,73]. 

### 4.2. Nurse Competencies

Competencies, as the core abilities by which nurses’ safely fulfil their roles and responsibilities [74], should be practically described for PRN medicines management and should form a guide for education. The European Federation of Nurses (EFN) Associations Guidelines describe the list of competencies for nurses responsible for general patient care in Article 31 of the Directive 2013/55/EU [75], and suggests its consideration in European nursing curricula. In Committee Competences A, D, and E of the professional qualifications directives (Directive 2013/55/EU and 2005/36/EC), the EFN Competence 6, “nursing care education and training”, indicates the recommended content of safe management of medicines and prescribing [76] (p. 24) as well as a set of student outcomes related to medicines management, as follows:

- Pharmacology knowledge;
- Medicine typologies;
- Pharmacokinetics;
- Administration method/dosage, interactions, circumstances that modify medicines management, contraindications, ADRs;
- Safe medicine management, and medication administration skills;
- Taking into account patient characteristics such as age, pathology and health condition in medicines management [76] (pp. 26–27);
- Taking a standardized medication history for medication reconciliation and detecting probable discrepancies [77].

With respect to nurse prescribing, as is in line with PRN medicines management, the EFN suggests the inclusion of learning outcomes that focus on the management of healthcare products linked to nursing care, and integrating the prescribing process into the nursing care process [76] (p. 27). 

As such, for the purposes of this discussion we have divided nurse competencies for PRN medicines management into the categories of “knowledge and skills”, “independence and interdependence in decision making”, and “monitoring and follow up”, all of which are described below and summarized in Table 1. 

#### 4.2.1. Knowledge and Skills

Measures to prevent medication errors and improve relevant aspects of patient safety are central elements in the competency-based education of medicines management [78]. These measures are characterized by integrated and comprehensive education throughout the undergraduate program, giving consideration to students’ learning styles and encouraging the students to self-regulate and improve their own learning [79,80]. Experience gained by nursing students during their basic education has long-lasting influence on how they perceive their role in medicines management, underpinning the need for well-established educational strategies [81]. 

Moreover, nursing students should be equipped with clinical knowledge and skills on how to use their theoretical knowledge about medication at the patient’s bedside [82], and, for instance, how to select appropriate medications for PRN [83]. As PRN medicines management depends on both teamwork and system-related factors, nursing students should also have knowledge of inter-professional communication, the complexity of care processes and relevant organizational structures such as guidelines and policies [81,84]. 

The essential concepts for PRN medication training are the 10 “rights” of medicines management: right patient, right reason, right drug, right route, right time, right dose, right form, right action, right documentation and right response [85]. In addition, in all cases of PRN medication, written guidelines and procedures for administering PRN medication should be verified in advance, including the following details:

- Circumstances affecting the use of PRN medications, including clinical and laboratory parameters;
- Doses, including the exact time and the maximum dose in a 24-hour period;
- Evaluation of the effectiveness of PRN medications after a determined time period [18];
- Monitoring patients for ADRs of the PRN medicine [28,86].

During training in medication reconciliation, which often includes PRN medications, sufficient opportunities should be provided for nursing students to observe and practice PRN in an interdisciplinary context, to become familiar with the multidisciplinary interactions involved in medicines management and the healthcare setting’s medication policy [87,88,89]. 

Preparation of nursing students to safely perform PRN medicines management requires the incorporation of innovative teaching methods [90]. For instance, digital technologies such as offline/online computer-based methods help with improving more medication skills than traditional education methods [91]. Watching video footage of medicines management and performing group discussions as problem-based learning help make students aware of factors contributing to medication errors [87]. Equally significant, simulations with high fidelity levels in the simulation laboratory and the use of tools such as medDISPENSE as the automated dispensing system and the Personal Digital Assistant (PDA) for digital recordings of the medication process are effective resources to reduce medication errors by nursing students [21]. Overall, the integration of educational methods consisting of simulation, group discussions and provision of feedback, case scenarios and problem-solving are a key step forward in this aspect of nurse education [92,93]. This “nursing case-based learning” helps to develop critical thinking abilities, based on situations in which decisions for safe medication practice should be made [94,95]. 

Clinical instructors should use practical examples to link classroom and clinical settings, improve medication calculation and medication administration skills and enhance the associated clinical judgment through reflection and discussion [81]. In clinical practice situations, supervision and role models during clinical rotations can help reduce medication errors by nursing students [96]. Role-play simulation that involves engaging nursing students in administering medications, observing their peers and/or causing interruptions and distractions improves their understanding of unfamiliar and challenging situations that require cooperation, teamwork and collaboration between nursing staff to reach the safe outcome of the medication process [97]. 

#### 4.2.2. Independence and Interdependence in Decision Making

Nursing students need to be competent to make independent clinical decisions on PRN medicines management, based on patient requests and their own clinical judgment, and including structured monitoring for adverse side effects [41]. They should examine their own thinking process and be prepared for unexpected and complex incidents in the medication process [98]. As a priority, they should be educated to take responsibility for nurse-led care initiatives and assess the effects of their own role on patients’ overall wellbeing and care outcomes [99]. 

Nursing students in clinical practice are not usually permitted to administer medication independently, but decision-making skills for safe PRN medicines management in high-risk and error-prone situations can be practiced in controlled situations and under the supervision of experienced nurses [92]. Nursing staff can act as mentors to coordinate learning opportunities for students to cope with real clinical situations that can cause anxiety, stress and distraction, helping to socialize them into their professional role and improving their capacity for evaluation and making clinical decisions through performing a check on the suitability of the PRN medications in relation to a changed pharmacokinetic status of the patient [78,90,100,101]. 

PRN medicines should be administered in conjunction with formal, structured checks for adverse effects [41]. Double checking should be practiced to enhance the safety of the medication process and prevent medication errors [102,103], along with other strategies such as reduced distractions, improved lighting and minimized noise levels within the environment when making high-risk medication decisions [104].

While the prescription and administration of PRN medications is the result of close nurse–patient collaboration and decision-making, the participation of other healthcare professionals involved in the medication process, such as physicians and pharmacists, is required for making the best decisions on the selection of PRN medications. Therefore, nursing students should receive training on accessing communication tools and contributing proactively to the decision-making process alongside other healthcare providers [105,106,107]. They should be educated to value the skills and responsibilities of other team members and respect their unique contributions in making decisions on medication safety [108]. The provision of interprofessional education, such as team-based learning, to students of medicine, nursing and pharmacy, aimed at safe prescribing, dispensing and administering of medicines [108,109,110,111], enhances competencies for safe PRN medicines management and reduces medication errors. 

Information technology has transformed decision-making processes in medicines management [112], particularly in the domains of documentation of nursing assessment, timing and dosage of PRN medications, adherence to practice guidelines, patterns of PRN medication prescription and administration, and monitoring of patient responses to medications [113]. Medicines management systems such as barcoding, electronic medical records, as well as wheeled workstations devised to reduce the potential for medication errors by giving nurses access to real-time information before making crucial decisions for medicines management, should be incorporated into nursing education goals [114].

#### 4.2.3. Monitoring and Follow-Up

A frequently overlooked aspect of nursing education is improving students’ competencies in assessing the patient’s care outcome during and after the medication process, and using the relevant data in patient care [83]. In PRN medicines management, the responsibility of nurses to ensure the safety of patients through appropriate follow up and monitoring during and after the medication process is doubled, due to the voluntary nature of medication prescription and administration based on the patient’s request [34]. 

Potential medication abuse by patients and the symptoms of addiction are further reasons for careful monitoring and follow up by nurses [115]. Nursing students should practice monitoring patients, with an emphasis on developing an understanding of how failures to assess and monitor patients during PRN medication prescriptions and administration constitute medication errors that need reporting [8,116].

In addition, the risk of unmonitored medication side effects and ADRs in healthcare settings during PRN medicines management is high, and should be highlighted during nursing education [28]. Nursing students should be made familiar with the use of available monitoring tools in order to assess and report ADRs as part of PRN prescription and administration. For instance, the medication monitoring instrument, the Adverse Drug Reaction Profile (ADRe), emphasizes nurses’ professional collaboration in multidisciplinary interventions and provides a structured method for monitoring, detecting and ameliorating probable ADRs [28,40,41,117]. This profile can be completed by nurses or carers alongside PRN medicines management, through the assessment of the patient’s health conditions, highlighting possible and probable ADRs on a single screen or sheet of paper, which is then passed to the pharmacist and physician. This informs a medication review process aiming at changing medications or their doses [41,86,118].

## 5. Conclusions

Nurses play a key role in PRN medicines management in terms of the preservation of patient safety, the prevention of medication errors and the improvement of patient outcomes. Nursing students are well-placed to learn about and practice how to safeguard PRN medicines management by considering patient participation, theoretical and practical knowledge and skills, independent decision-making, inter-professional collaboration, monitoring and follow-up. In addition to the emphasis on the theoretical principles of PRN medicines management, attention should be paid to its practical considerations, using appropriate educational strategies as outlined above. Given the paucity of literature on this topic, however, there remains a pressing need for original research to help us improve our understanding of the barriers to and the facilitators of effective PRN medicines management by nurses in clinical practice.

## Figures and Tables

**Table 1:** Summary of nurse competencies for safe PRN medicines management.

Competency Category	Principles	Educational Strategy
Knowledge and skills	Selection of appropriate medications; Team work and interprofessional communication; Guidelines and policies; Rights of medicines management; Condition, dose and effectiveness of medications; Medication-taking history; Medication calculation and administration	Provision of opportunities to practice at bedside; Computer-based methods; High-fidelity simulation; Problem-based learning; Reflection and discussion; Supervision and role model; Case study learning
Independence and interdependence in decision making	Making independent clinical decisions; Feeling of responsibility; Linking between interventions and patient’s well-being; Double checking; Nurse-patient collaboration; Involvement of healthcare staff; Use of interprofessional communication tools; Valuing and respecting skills and responsibilities; Use of information technology	Self-reflection and criticism; Provision of controlled decision-making situations; Being mentored by clinical nurses; Provision of safe environment for the medication process; Interprofessional education;
Monitoring and follow up	Assessing care outcomes before, during and after medication; Monitoring abuse; Use of monitoring tools and profiles;	Monitoring patients for the signs and symptoms of known adverse side effects and reporting anything that may be related to the PRN medicine to pharmacists or prescribers.

ADR: adverse drug reaction.
